# Laparoscopic surgery versus robot-assisted surgery for choledochal cyst excision: A systematic review and meta-analysis

**DOI:** 10.3389/fped.2022.987789

**Published:** 2022-10-26

**Authors:** Ke Zhang, Difang Zhao, Xiaolong Xie, Wentao Wang, Bo Xiang

**Affiliations:** ^1^ Department of Pediatric Surgery, West China Hospital, Sichuan University, Chengdu, China; ^2^ Department of Surgical Room, West China Hospital, Sichuan University, Chengdu, China

**Keywords:** choledochal cyst excision, laparoscopic surgery, robot-assisted surgery, systematic review, meta-analysis

## Abstract

The aim of this following study is to systematically review and analyze the published data comparing laparoscopic surgery and robotic assisted surgery for choledochal cyst excisions through the metrics of operative time, length of hospital stay and postoperative outcome. PubMed, Web of Science, Embase, Ovid, and the Cochrane Library databases were combed through and data was retrieved from the timespan between January 1995 and October 2021. The primary measures included operative time, intraoperative bleeding, hospital stay, and postoperative complications. Quality and risk of bias were assessed using the Newcastle-Ottawa Quality Assessment Scale. Making use of random-effects models, we pooled the odds ratios (ORs) and mean differences (MDs) with 95% confidence intervals (95% CIs). Six studies comprising a total 484 patients who had undergone either laparoscopic surgery [307 (63.43%) patients] or robot-assisted surgery [177 (36.57%) patients] were included in this analysis. Three of the articles involved adults while the other three involved children. All of the studies were published after 2018 and were retrospective case–control studies. Patients undergoing robotic surgery had a shorter hospital stay (MD, 0.95; 95% CI, 0.56 to 1.35; *p* < 0.00001) and a longer operative time (MD, −57.52; 95% CI, −67.17 to −47.87; *p* < 0.00001). And there was no significant discrepancy in complications between the two groups. Compared to laparoscopic surgery, robot-assisted surgery is associated with a shorter hospital stay, scores highly in terms of both safety and feasibility, however it also results in a longer operative time. And the two procedures have the same short- and long-term results.

## Introduction

Choledochal cysts (CCs) are extremely rare malformations found in bile ducts involving dilatation and pancreaticobiliary maljunction (1). They often lead to symptoms such as abdominal pain, vomiting, jaundice, and fever (2). Without effective treatment, patients with choledochal cysts may experience cyst perforation, recurrent pancreatitis, canceration, and even severe cholestasis, which itself eventually results in liver cirrhosis, portal hypertension, and ultimately liver failure (3).

The main treatments for choledochal cysts are the complete resection of the cyst, cholecystectomy and Roux-en-Y choledochojejunostomy, which traditionally have been performed as open procedures (1–3). The laparoscopic approach towards treating choledochal cysts has gradually joined these previously mentioned procedures as a mainstream method over the last decade, ever since Farello et al. executed the very first laparoscopic choledochal cyst resection with a hepaticoenterostomy successfully on a girl of the age of six in 1995 (4). However, due to their intense technical demands laparoscopic approaches have still not achieved widespread usage, especially the hepaticoenterostomy which requires a certain learning curve. At the same time, robotic assisted surgery has been suggested as an alternative method for pediatric choledochal cyst excision, as the first robot-assisted choledochal cyst resection in children was reported by Woo et al. in 2006 (5, 6). Robot-assisted surgery has several features that give it an advantage. Examples of these include its operability and accuracy being enhanced relative to laparoscopic surgery due to its three-dimensional imaging and the flexible design of its simulation manipulator (7).

To our knowledge few systematic review or meta-analysis has as of yet been published comparing laparoscopic surgery and robotic assisted surgery for choledochal cyst excisions to determine which is the preferential treatment. The aim of the present study is to systematically review and analyze the published data comparing laparoscopic surgery and robotic assisted surgery for choledochal cyst excision regarding operative times, length of hospital stay and postoperative outcomes.

## Methods

### Information source and search strategy

This systematic review/meta-analysis was performed using Medline, PubMed, Web of Science, Embase, Ovid, and the Cochrane Library databases with articles within the time period from January 1995 to October 2021 being sought after. The search terms were: “choledochal cyst or congenital biliary dilatation”, “laparoscopy”, “robot or da Vinci”. The search was limited to the English language only. Furthermore, reference lists of relevant papers were also explored for any other studies of interest. The study was registered with the PROSPERO database (CRD42021283740).

### Inclusion and exclusion criteria

Studies were considered eligible for inclusion as long as they met the following criteria: (1) they conducted a comparative study of laparoscopic surgery and robot-assisted surgery that were definitely done to treat a choledochal cyst; (2) the outcome data reported was available, and (3) the study format was full text only; editorials, case reports, abstracts, and conference presentations were left out. If studies did not meet the inclusion criteria, they were not included.

### Study selection and data collection

All of the study titles and abstracts selected for further study were cross-checked by the two independent authors (KZ and XXL). Eligible studies that met the inclusion criteria were then retrieved. The relevant data from the included studies—specifically their study characteristics and outcomes—was then extracted by those two authors separately. Complete agreement was necessary at each stage of the study selection process and if a discrepancy occurred, a third investigator would step in to resolve the matter (DFZ).

In each of the studies, the following information was deemed notable: the lead author's name, country, year of publication, study type, follow-up time, mean age and number of patients in each group, cystic diameter, and outcomes (e.g., operative time, enteral feeding time, length of hospital stay, as well as postoperative complications including bile leaking, adhesive intestinal obstruction, bleeding, cholangitis, bile reflux/gastritis, and reoperation rates). Throughout the course of this study, if there was missing data like the standard deviation, it would be calculated based on formulas from the Cochrane handbook.

### Quality assessment

In order to assess the quality attributes and to help eliminate any risk of bias in the nonrandomized studies chosen in this meta-analysis, the Newcastle-Ottawa Quality Assessment Scale (NOS) was employed. Each study was assessed according to three criteria in a sort of “star system”: selection, comparability, and ascertainment of exposure. Eight items were covered in this scale: four points could be scored for selection; two points, for comparability; and three points, for exposure. A maximum of one star for each numbered item within the selection and exposure categories could be awarded to each study. For the comparability categories, there was a maximum of two stars which could be rewarded. The higher the score was, the higher the quality of the study. Studies with 1–3 stars were deemed to be “low quality,” while with 4–6 stars ones were considered of “moderate quality,” and studies with 6–9 stars “high quality” respectively.

### Statistical analysis

To pool all of the data the RevMan 5.4 statistical software, which was updated by the Cochrane Library for Systematic Reviews, was employed. This produced forest plots, funnel plots, pooled odds ratio (OR), pooled the mean difference (MD), and confidence intervals (CIs) found in this study. Regarding the dichotomous variables, the Mantel–Haenszel method assisted in computing the OR. As to the continuous variables however, the inverse variance method assisted in the calculation of the MD. The CI was set at 95%, and a value of *p *< 0.05 was considered statistically significant. In order to assess the heterogeneity of the study, the *I*^2^ statistic—representing the percentage of between-study variation—was measured. In the case that the total number of publications included for each outcome exceeded 10, funnel plots were established so as to attempt to indicate any evidence of potential.

## Results

**Figure 1:**
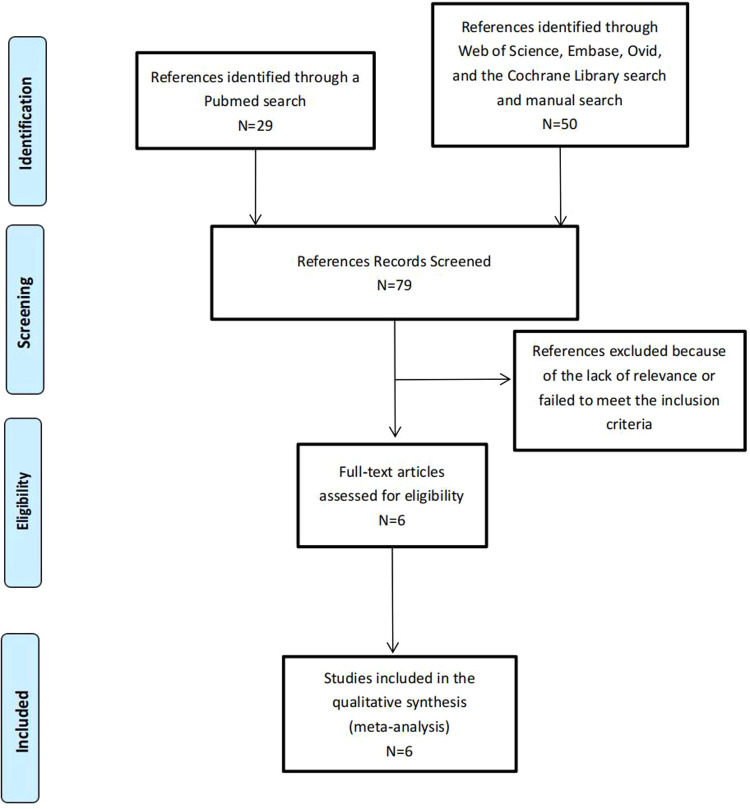
The flowchart of this systematic review and meta-analysis.

Altogether, there was a total of seventy-nine articles identified, and these included two studies which were manually drawn from the references found in other studies. Seventy-three articles were left out due to either lack of relevance or a failure to meet the inclusion criteria set out. The six remaining studies included a total of 484 patients, of which 307 underwent laparoscopic surgery (63.43%) and 177 underwent robot-assisted surgery (36.57%), and were included in the analysis (8–13) (Figure 1). The characteristics of the included studies are summarized in Table 1. Five studies all used robots to complete cyst resection and choledochojejunostomy while Koga H et al. completed cyst resection by laparoscopic procedures and choledochojunostomy by robotic procedures (10). Three of the articles involved adults (8, 9, 12) and the other three involved children (10, 11, 13). All of the studies were published after 2018 and were retrospective case–control studies (8–13). The NOS scale was used to assess study quality among the six studies: all six studies were deemed to be of high quality and no study was of low quality.

**Table 1:** Basic characteristics of the enrolled studies.

	Year	Country		Design of study	Year of publication	Total number of patients (LG/RG)	Women, No. (%) (LG/RG)
Hongeun Lee	2018	Korea		Retrospective	2004–2016	67 (49/18)	43 (87.8%)/18 (100%)
Jang Hun Han	2018	Korea		Retrospective	2014–2017	56 (34/22)	27 (79.4%)/22 (100%)
Hiroyuki Koga	2019	Japan		Retrospective	2017–2019	37 (27/10)	-
Xiaolong Xie	2020	China		Retrospective	2015–2018	145 (104/41)	79 (75.96%)/31 (75.61%)
Jong Hwi Yoon	2021	Korea		Retrospective	2005–2018	39 (23/16)	20 (87.0%)/13 (81.3%)
Shui-qing Chi	2021	China		Retrospective	2014–2019	140 (70/70)	48 (68.57%)/48 (68.57%)
	Age (years) mean (SD) (LG/RG)		Cystic diameter (cm) (LG/RG)			Follow up (months) (LG/RG)	Primary outcomes
Hongeun Lee	36.57 ± 10.84/36.17 ± 13.33		-			-	Duration of surgery, LOS, Complications
Jang Hun Han	37.5 ± 11.6/35.3 ± 11.05		3.14*5.23/3.14*5.04			5–100/5–100	Duration of surgery, LOS, Complications
Hiroyuki Koga	5.2 ± 3.8/5.6 ± 3.4		-			36/20	Duration of surgery, LOS, Complications
Xiaolong Xie	2.33 (0.73–4.42)/4.0 (2.54–6.46)		3.78 ± 2.39/3.18 ± 1.65			-	Duration of surgery, LOS, Complications
Jong Hwi Yoon	34.3 ± 11.2/37.0 ± 10.7		0.98 ± 0.19/1.15 ± 0.43			-	Duration of surgery, LOS, Complications
Shui-qing Chi	36.21 ± 32.80/34.00 ± 27.71		-			-	Duration of surgery, LOS, Complications

LG, laparoscopic procedures group; RG, robot-assisted procedures group; LOS, length of hospital stay; SD, standard deviation.

### Operative time

**Figure 2:**
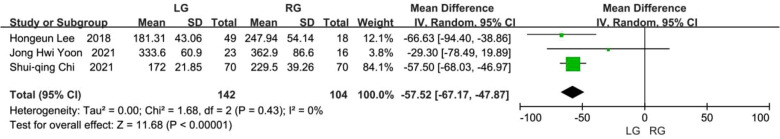
Operative time.

Six studies reported on the operative time with the mean operative time and standard deviation (8–13), while three were excluded following heterogeneity analysis (9–11). The three remaining studies included a total of 246 patients, and of these 142 underwent laparoscopic surgery and 104 underwent robot-assisted surgery (8, 12, 14). The pooled mean difference showed that the operative time of robot-assisted surgery was longer than the laparoscopic surgery and that the difference was statistically significant (MD, −57.52; 95% CI, −67.17 to −47.87; *p* < 0.00001) (Figure 2).

### Intraoperative bleeding

**Figure 3:**
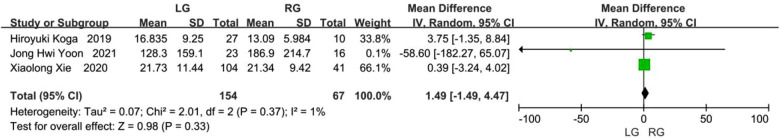
Intraoperative bleeding.

Five studies provided data on the intraoperative bleeding (8, 10, 11–13), however two of these articles were excluded following heterogeneity analysis (8, 13). The three remaining studies included 221 patients, of which 154 underwent laparoscopic surgery and 67 underwent robot-assisted surgery (10–12). The difference was not statistically significant between these two groups (MD, 1.49; 95% CI, −1.49 to 4.47; *p* = 0.33) (Figure 3).

### Bile leakage

**Figure 4:**
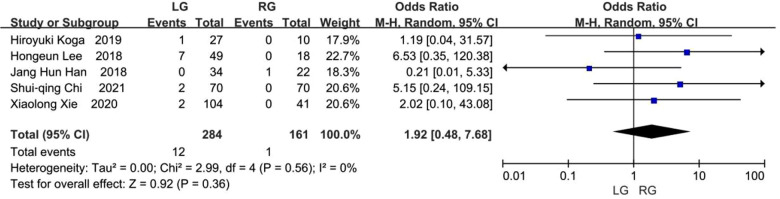
Bile leak.

Six studies provided data on bile leakage (8–13), while one article was excluded following heterogeneity analysis (12). As reported in the five remaining studies with a total of 445 patients (8–11, 13), 12 (4.23%) of the 284 patients in the laparoscopic group and 1 (0.62%) of the 161 patients in the robotic group experienced bile leakage. No statistically significant difference existed between the two groups, but the results favored the robotic group (OR, 1.92; 95% CI, 0.48 to 7.68; *p* = 0.36) (Figure 4).

### Wound infection

**Figure 5:**
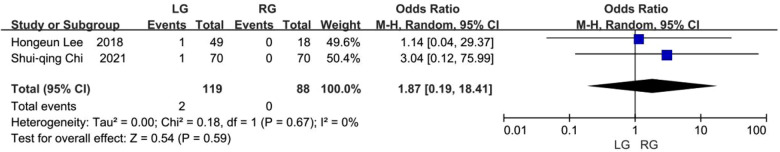
Wound infection.

Two studies provided data on wound infection (8, 13). Wound infection occurred in 2 (1.68%) of the 119 patients in the laparoscopic group and none of the 88 patients in the robotic group. The pooled results showed that no difference was statistically significant between the two groups, but the results favored the robotic group (OR, 1.87; 95% CI, 0.19 to 18.41; *p* = 0.59) (Figure 5).

### Hospital stay and analgesia treatment

**Figure 6:**
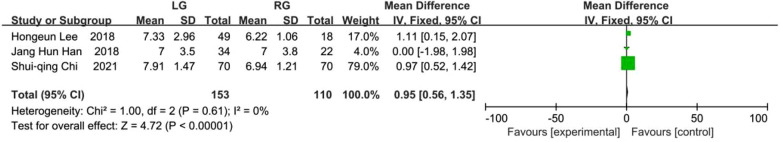
Hospital stay.

Six of the studies delivered data regarding the length of hospital stay (8–13), while 3 articles were excluded following the heterogeneity analysis (10–12). The 3 remaining studies included 263 patients, and of these there were 153 who underwent laparoscopic surgery and 110 who underwent robot-assisted surgery (8, 9, 13). The pooled results showed that the hospital stay of robot-assisted surgery was shorter than that of laparoscopic surgery and the difference was considered statistically noteworthy (MD, 0.95; 95% CI, 0.56 to 1.35; *p* < 0.00001) (Figure 6). Only one of the six studies described analgesia treatment after surgery and the results showed that postoperative pain medication usage was signifcantly lower in robotic surgery than in laparoscopic surgery (*p* < 0.001) (11).

### Bleeding

**Figure 7:**
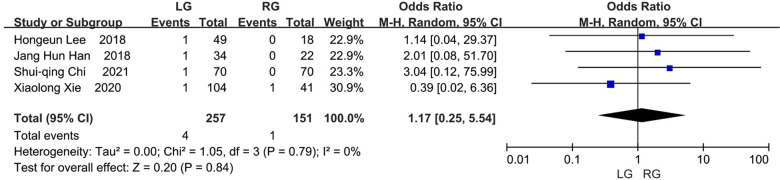
Bleeding.

Four studies delivered data regarding the number of patients suffering from bleeding, and this occurred in 4 (1.56%) of the 257 patients in the laparoscopic group and 1 (0.66%) of the 151 patients in the robotic group (8, 9, 11, 13). The difference was not statistically significant between these two groups and the results favored the robotic group (OR, 1.17; 95% CI, 0.25 to 5.54; *p* = 0.84) (Figure 7).

### Cholangitis

**Figure 8:**
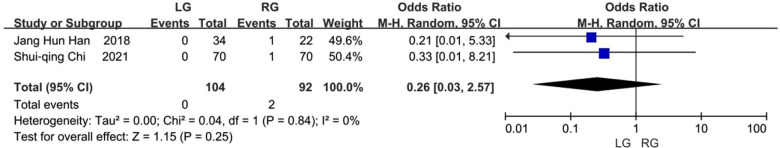
Cholangitis.

Two studies reported on the incidence of cholangitis after biliary reconstruction (9, 13). In these two studies, 2 (2.17%) of the 92 patients in the robotic group and none of the 104 patients in the laparoscopic group experienced cholangitis. No significant difference existed between the groups, but the results favored the laparoscopic group (OR, 0.26; 95% CI, 0.03 to 2.57; *p* = 0.25) (Figure 8).

### Anastomotic stricture

**Figure 9:**
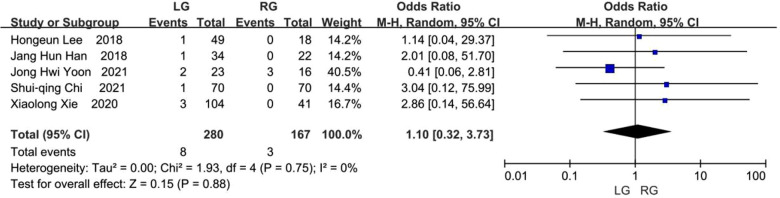
Anastomotic stricture.

Occurrences of anastomotic stricture were reported on in five studies (8, 9, 11–13). Anastomotic stricture occurred in 8 (2.86%) of the 280 patients in the laparoscopic group and 3 (1.80%) of the 167 patients in the robotic group. There was no significant statistical deviation between the two groups, but the results favored the robotic group (OR, 1.10; 95% CI, 0.32 to 3.73; *p* = 0.88) (Figure 9).

### Adhesive intestinal obstruction

**Figure 10:**
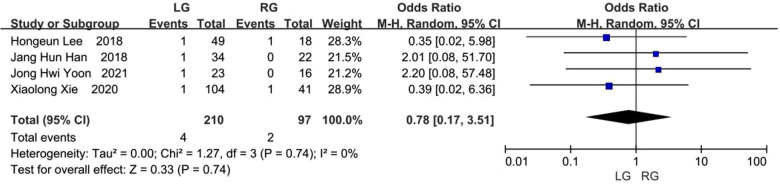
Adhesive intestinal obstruction.

Four studies assessed the rate of occurrence of adhesive intestinal obstruction (8, 9, 11, 12), and this took place in 4 (1.90%) of the 210 patients in the laparoscopic group and 2 (2.06%) of the 97 patients in the robotic group. The pooled OR indicated that there was no statistical difference found in adhesive intestinal obstruction among these two groups, but that the results favored the laparoscopic group (OR, 0.78; 95% CI: 0.17 to 3.51; *p* = 0.74) (Figure 10).

### Residual cysts

**Figure 11:**
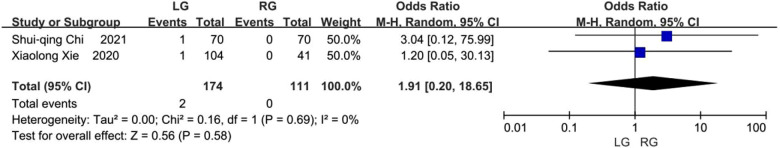
Residual cyst.

Two studies reported on the incidence of residual cysts after the choledochal cyst excision procedure (12, 13). In the two studies, 2 (1.15%) of the 174 patients in the laparoscopic group and none of the 111 patients in the robotic group experienced residual cysts. No significant difference existed between the laparoscopic and robotic groups, but the results favored the robotic group (OR, 1.91; 95% CI, 0.20 to 18.65; *p* = 0.58) (Figure 11).

### Biliary stones

**Figure 12:**
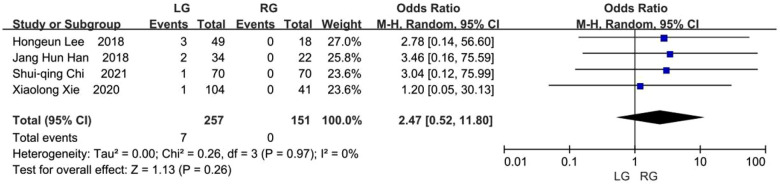
Biliary stones.

Four studies reported on the incidence of biliary stones (8, 9, 11, 13). Biliary stones occurred in 7 (2.72%) of the 257 patients in the laparoscopic group and none of the 151 patients in the robotic group. No significant statistical difference was found between these two groups, but the results favored the robotic group (OR, 2.47; 95% CI, 0.52 to 11.80; *p* = 0.26) (Figure 12).

### Reoperation rates

**Figure 13:**
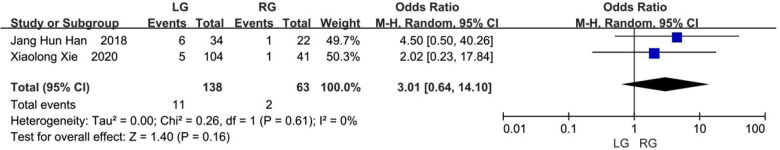
Reoperation rates.

Two studies reported on the need for secondary operation rates (9, 11). In the laparoscopic group, 11 (7.97%) of the 138 patients needed reoperation surgery, compared to 2 (3.17%) of the 63 patients in the robotic group. There was no statistically significant difference, as revealed by the meta-analysis (OR, 3.01; 95% CI, 0.64 to 14.10; *p* = 0.16) (Figure 13).

There were two additional articles which reported on enteral feeding time and hospitalization expenses respectively, but these were excluded following the heterogeneity analysis.

## Discussion

The most common method of treatment for choledochal cysts tends to be the complete resection of the cyst using a Roux-en-Y hepaticojejunostomy, and this has been traditionally performed as an open procedure (1). Along with the increasing focus on aesthetic considerations when considering treatment, laparoscopic approaches in hepatobiliary surgery have become the inevitable result. From when the first laparoscopic choledochal cyst excision took place in 1995 (4), studies on this approach have consistently reported that laparoscopic surgery scores highly both in terms of safety and feasibility in the treating of choledochal cysts. As laparoscopy is minimally invasive, leading to a cosmetically enhanced recovery as well as providing better vision of the deep anatomic structures compared with open approaches, it possesses significant advantages (14). However, the drive towards using laparoscopic approaches to perform choledochal cyst excisions has been sluggish, mainly due to the technical complexities of these procedures. They require a considerable learning curve, with hepaticojejunostomy being a notable example of this. In addition to this, there are several other limitations, such as: necessary usage of straight rigid instruments within a tight working space, a limited degree of freedom to work within, instability of the camera platform with two-dimensional imaging, and non-ergonomic instruments. Robot-assisted surgery though offers several technical advantages over laparoscopic surgery, and these include: high-quality three-dimensional imaging, free-moving multi-joint forceps and image stabilization. As a result, laparoscopic surgery's learning curve can be shortened. Despite this, a serious lack of data and large-scale sample cases which compare the safety and effectiveness of these two surgical methods for choledochal cyst excisions, remains an ever-present issue.

This systematic review and meta-analysis compares the outcomes of laparoscopic surgery and robot-assisted surgery, using studies in the literature as a basis for review. It was indicated by the findings that robot-assisted surgery resulted in a hospital stay of shorter duration and longer operative time. One possible reason why the operative time of robotic surgery is significantly longer than that of laparoscopic surgery is that the operative time in robotic surgery includes both the docking time and instruments replacement time. Xie et al. reported on the learning curve for the robot-assisted choledochal cyst excision and Roux-en-Y hepaticojejunostomy with the da Vinci surgical system methods and came to the conclusion that it was 14 cases (15). With improvement in the learning curve, the installation time decreases gradually. A few studies also reported that robotic surgery takes less time than laparoscopic surgery (9–11). What's more, robot-assisted approaches result in a shorter hospital stay, which might be caused by the substantial improvements in visibility and manipulation through the use of 3D imaging, tremor filters, and articulated instruments (16). The imaging of 3D can more clearly reveal the deep anatomical structure and the doctors can adjust the lens depth and angle according to their own habits and requirements. The simulation manipulator of the robotic surgery is highly flexible, simulating the translation, bending, opening and closing, rotation and other operations of the human hand. It can even rotate 540° to accurately grasp, free, cut and sew. It also has the functions of eliminating vibration and motion calibration. Each step of the robotic surgery has less interference on the intestine leading to less interference. As for the enteral feeding time, only two of the six articles included reported on this, but these were excluded following heterogeneity analysis. Thus more reports are needed to verify the impact of these two methods on postoperative enteral feeding time.

The reports on complications after laparoscopic or robotic choledochal cyst resections yielded differing results. Xie et al. reported that complications found in laparoscopic procedures were of a higher rate than those found in the robot-assisted procedures, but also that there was no meaningful difference found between the two groups overall (11). And robotic surgery can remove the tissue of cyst to the maximum extent which the distal end of cyst can be finely dissected down to the pancreatic segment and the proximal end is closed to the hilar bile duct. Hiroyuki et al. discovered that robotic surgery was positively correlated with enhanced postoperative outcomes when compared with laparoscopic surgery, such as less estimated blood loss, less time needed for the drainage tube insertion and a shorter duration for bowel sound return (10). Markar et al. undertook a systematic review and demonstrated that the occurence of anastomotic stricture in the robotic Roux-en-Y gastric bypass procedure within the laparoscopic group was a significantly reduced (17). Shui-qing Chi et al. reported that robotic surgery had obvious advantages for cyst excision and could provide a clearer view of hepatic duct anatomy which had encountered difculties in cyst dissection and was related to enhanced intraoperative and short-term postoperative outcomes in comparison to laparoscopic-assisted surgery (13). Robot surgery was not a single link that improves the quality of choledochal cyst excision which was improved as a whole with the help of the clarity, flexibility and stability of the robot operating system. The sharp cutting of the curvature of the electric shear protected the blood supply of the bile duct and intestinal to be anastomosed, reduced the damage to the blood supply of the bile duct and intestinal and improves the healing ability of the anastomosis. The mucosa to endothelium and small diameter of hepaticojejunostomy could be completed without difficulty. Abnormal blood vessels and bile ducts could also be clearly found and easier and more accurate to be dissected. In this systematic review and meta-analysis, laparoscopic surgery was found comparable to that of robot-assisted surgery as relates to common postoperative outcomes (e.g., cholangitis, bile leak, anastomotic stricture, bleeding, and reoperation rates). Despite there being no difference in postoperative complications found between laparoscopic and robotic surgery, Shui-qing Chi et al. reported that enhanced clarity of the hepatic duct anatomy was provided by the robotic system compared to traditional laparoscopy. A clear surgical field of vision assists surgeons in treating the lesions in a more accurate and thorough way, and can help to avoid or at least limit complications associated with surgical procedures to a certain degree (13).

At the same time, robotic surgery certainly also has its share of defects. Firstly, in general the cost of robotic procedures is significantly higher than that of other techniques. In fact, in China the cost of robotic surgery is approximately 20–40 thousand RMB (3,094–6,188 US dollars, according to the exchange rate in October 2021) and this is higher compared with open and laparoscopic methods. Two of the six articles included in this review reported on hospitalization expenses, but were excluded following heterogeneity analysis. Besides this, the da Vinci surgical system lacks a function providing tactile feedback, and as such the operator cannot directly feel the mechanical feedback when separating, suturing, and knotting. However, with improvements in the learning curve, visual feedback through hand-eye coordination will eventually make up for this absence in tactile feedback.

This study has some notable limitations. First, all included articles were retrospective analyses. Second, the amount articles were relatively small in number. Future large and multicenter prospective studies consisting of a greater population of patients and follow-ups over a longer-term are needed to further assess and compare the safety and feasibility of laparoscopic and robotic surgery. However, this review make use of meta-analysis in systematically reviewing and analyzing the published data comparing laparoscopic surgery and robotic assisted surgery for choledochal cyst excisions in terms of clinical outcomes.

## Conclusion

Compared to laparoscopic surgery, robot-assisted surgery is associated with a shorter hospital stay, scores highly in terms of both safety and feasibility, however it also results in a longer operative time. And the two procedures have the same short- and long-term results.

## Data Availability

The raw data supporting the conclusions of this article will be made available by the authors, without undue reservation.
